# Large-scale field trial of attractive toxic sugar baits (ATSB) for the control of malaria vector mosquitoes in Mali, West Africa

**DOI:** 10.1186/s12936-020-3132-0

**Published:** 2020-02-14

**Authors:** Mohamad M. Traore, Amy Junnila, Sekou F. Traore, Seydou Doumbia, Edita E. Revay, Vasiliy D. Kravchenko, Yosef Schlein, Kristopher L. Arheart, Petrányi Gergely, Rui-De Xue, Axel Hausmann, Robert Beck, Alex Prozorov, Rabiatou A. Diarra, Aboubakr S. Kone, Silas Majambere, John Bradley, John Vontas, John C. Beier, Günter C. Müller

**Affiliations:** 1 Malaria Research and Training Centre, Faculty of Medicine, Pharmacy and Odonto-Stomatology, University of Sciences, Techniques and Technology of Bamako, BP 1805, Bamako, Mali; 2 Department of Zoology, Tel Aviv University, 61500 Tel Aviv, Israel; 3 Department of Microbiology and Molecular Genetics, IMRIC, Kuvin Centre for the Study of Infectious and Tropical Diseases, Faculty of Medicine, Hebrew University, 91120 Jerusalem, Israel; 4 Department of Public Health Sciences, Miller School of Medicine, University of Miami, Miami, FL 33136 USA; 5 SNSB-Zoologische Staatssammlung, 81247 Munich, Germany; 6 Anastasia Mosquito Control District, 120 EOC, St. Augustine, FL 32092 USA; 7 Pan-African Mosquito Control Association, P.O. Box 54840-00200, Nairobi, Kenya; 8 MRC Tropical Epidemiology Group, London School of Hygiene and Tropical Medicine, London, WC1E 7HT UK; 9 Institute of Molecular Biology and Biotechnology, Foundation for Research and Technology-Hellas, 70013 Heraklion, Greece; 10 Pesticide Science Laboratory, Department of Crop Science, Agricultural University of Athens, 11855 Athens, Greece

**Keywords:** *Anopheles gambiae*, ATSB, Vector control, Mali, Sugar feeding

## Abstract

**Background:**

The aim of this field trial was to evaluate the efficacy of attractive toxic sugar baits (ATSB) in Mali, where sustained malaria transmission occurs despite the use of long-lasting insecticidal nets (LLINs). ATSB bait stations were deployed in seven of 14 similar study villages, where LLINs were already in widespread use. The combined use of ATSB and LLINs was tested to see if it would substantially reduce parasite transmission by *Anopheles gambiae* sensu lato beyond use of LLINs alone.

**Methods:**

A 2-day field experiment was conducted to determine the number of mosquitoes feeding on natural sugar versus those feeding on bait stations containing attractive sugar bait without toxin (ASB)—but with food dye. This was done each month in seven random villages from April to December 2016. In the following year, in seven treatment villages from May to December 2017, two ATSB bait stations containing the insecticide dinotefuran were placed on the outer walls of each building. Vector population density was evaluated monthly by CDC UV light traps, malaise traps, pyrethrum spray (PSCs) and human landing catches (HLCs). Female samples of the catch were tested for age by examination of the ovarioles in dissected ovaries and identification of *Plasmodium falciparum* sporozoite infection by ELISA. Entomological inoculation rates (EIR) were calculated, and reductions between treated and untreated villages were determined.

**Results:**

In the 2-day experiment with ASB each month, there was a lower number of male and female mosquitoes feeding on the natural sugar sources than on the ASB. ATSB deployment reduced CDC-UV trap female catches in September, when catches were highest, were by 57.4% compared to catches in control sites. Similarly, malaise trap catches showed a 44.3% reduction of females in August and PSC catches of females were reduced by 48.7% in September. Reductions of females in HLCs were lower by 19.8% indoors and 26.3% outdoors in September. The high reduction seen in the rainy season was similar for males and reductions in population density for both males and females were > 70% during the dry season. Reductions of females with ≥ 3 gonotrophic cycles were recorded every month amounting to 97.1% in October and 100.0% in December. Reductions in monthly EIRs ranged from 77.76 to 100.00% indoors and 84.95% to 100.00% outdoors. The number of sporozoite infected females from traps was reduced by 97.83% at treated villages compared to controls.

**Conclusions:**

Attractive toxic sugar baits used against *Anopheles* mosquitoes in Mali drastically reduced the density of mosquitoes, the number of older females, the number of sporozoite infected females and the EIR demonstrating how ATSB significantly reduces malaria parasite transmission.

## Background

Malaria is one of the most devastating diseases in Africa where it is mostly transmitted by *Anopheles gambiae* sensu stricto (s.s.), *Anopheles coluzzii*, *Anopheles funestus*, and *Anopheles arabiensis* [1]. In 2017, the World Health Organization (WHO) estimated that there were 219 million cases of malaria globally [2], and that ‘no significant progress in reducing global malaria cases was made in recent years’ [2]. There is a clear need to develop and integrate new vector control strategies to further reduce malaria transmission [3–5]. Current vector control methods include the use of long-lasting insecticidal nets (LLINS), indoor residual spraying (IRS), and larval control [6–8]. Integrated vector management (IVM) seeks to optimize the use current interventions and to integrate them with new methods as they become available [6, 9] especially in problematic areas of sustained transmission despite the scale-up of LLINs or IRS [10–14].

Sugar-feeding is critical to the survival of African malaria vectors [15–18] and the availability of sugar sources in the local environment is a key regulator of mosquito population dynamics and, therefore, vectorial capacity [15, 16]. The attractive toxic sugar bait (ATSB) method is a mosquito control system that exploits the need for both male and female mosquitoes to take vital sugar meals [19, 20]. It comprises a bait, attractive to the species of interest (usually with a fruit or flower scent), and contains an oral toxin mixed with sugar as a feeding stimulant [21]. There are many different classes of oral toxins that can be used for ATSB, including carbamates, pyrethroids, neonicotinoids, spinosyns, borates, biopesticides and double-stranded RNA [21–26]. ATSB has been applied as a spray on vegetation and blossoms [21, 22, 27] and has also been incorporated into bait stations which can be hung walls of houses or inside cisterns and drains [22]. Excellent mosquito control has been achieved in small-scale experiments with ATSB in Israel where it was developed and extensively tested against numerous species [21, 23, 24–26]. The potential of ATSB to control mosquitoes was also demonstrated in successful field experiments in Mali [23, 28, 29] and in the USA [23, 30].

The current field trial in Mali tested the potential impact of ATSB by different criteria including vector densities, the rate of biting on humans, the proportion of ‘old’ females that passed at least three or more egg laying cycles, sporozoite infection rate, and EIR. An obvious advantage of ATSB is its function outdoors against exophilic *Anopheles* spp., which are not directly exposed to IRS and LLINs, and can be effective against insecticide resistance and the behavioural plasticity shown by *Anopheles* in response to the scale-up of indoor interventions [11–14]. It is expected that in the long-term integrated vector management (IVM) programme that combines both ATSB and currently used LLINs would substantially contribute to the reduction of malaria transmission.

## Methods

### Experimental villages and characterization of mosquito populations

In the Keyes Province of Mali (14.115, − 10.568611), West Africa 14 villages accessible by car, and within 10 km of the Niger River were selected in 2016 (see Additional file 1: Fig. S1) and baseline entomological surveys were conducted (unpublished). The dominant mosquito species were *An. coluzzii* and *An. gambiae* s.s. (unpublished data). In the field trial of ATSB in 2017, seven of the 14 villages, were allocated to treatment and seven were control villages. All of the villages had > 90% LLINs coverage and no other anti-mosquito interventions (including mass drug administration MDA), determined by a separate social science team inspecting homes and talking to residents. These 14 villages (Table 1) were randomly assigned to treatment and parallel control groups. Villages were assigned a random number in Microsoft Excel, odd numbers were assigned to the control group and even numbers to the treatment group.

**Table 1:** Map coordinates, population, and distance to the Niger river of control and experimental villages

Control villages	Latitude, longitude	Pop.	Dist. (km)	Treated villages	Latitude, longitude	Pop.	Dist. (km)
Balandougou	11.985750, − 8.51395	< 500	> 5	Krekrelo	11.98836, − 8.551460	< 500	> 5
Madina	12.05229, − 8.373600	< 500	< 5	Sirakele	11.95466, − 8.446560	< 500	> 5
Korea	12.04576, − 8.399230	< 500	> 5	Trekrou	12.068577, − 8.314414	> 500	< 5
Balala	11.96599, − 8.468310	> 500	> 5	Farabale	12.03865, − 8.424590	< 500	> 5
Cissebougou	12.09628, − 8.372850	> 500	> 5	Kignele	11.96737, − 8.397900	> 500	< 5
Nianguanabougou	12.15466, − 8.308260	> 500	< 5	Tiko	12.13444, − 8.396860	> 500	> 5
Trekrouba	12.073598, − 8.258717	> 500	< 5	Sambadani	12.14454, − 8.316880	> 500	< 5

*Pop* population, *Dist* distance to river

### Climatic conditions

The study area experiences tropical wet and dry seasons. The hottest months are March, April, and May (average daily maximum temperature is 32.4 °C in May). The average daily maximum temperature in the coldest month (December) is 25.1 °C. Total annual rainfall averages 1098.5 mm; the rainy season begins in May and peaks in August/September. The driest periods are late October through April [31].

### Malaria vector feeding rate on natural sugar versus attractive sugar bait stations (ASB)

In a 2-day experiment carried out in 2016, mosquitoes were collected in seven random villages every month with 10 UV CDC light traps (Model 512, John W. Hock Company, Gainesville, Florida, USA), set approximately 5 m away from 10 different houses in the center of the village, in a rough grid pattern, at least 10 m away from each other, for 12 h (from 18:00 to 06:00 h), from April to December 2016. Houses were chosen based on the willingness of the inhabitants to participate. The following day, two bait stations with ASB (no toxin) and with green coloured food dye (Tartrazine 19140, Stern, Natanya, Israel) were placed on every house in the village, hung 1.8 m above ground on an outside wall, and mosquitoes were collected repeatedly from the same location around these houses for 12 h with the same CDC UV light traps. Anthrone tests of mosquitoes captured prior to the 1st day of using bait stations showed the percentage of mosquitoes that were feeding on natural sugar sources. Mosquitoes caught by stained bait stations with dye marked bait inside, a night later showed the rate of daily feeding on baits, while the number of unstained yet anthrone positive mosquitoes from the same catch showed the number of mosquitoes that fed on natural sugar sources. For a visual demonstration of colour in the guts of *An. gambiae* sensu lato (s.l.), see [32].

### Testing for sugar and bait feeding

The sugar content in the gut of a random subset of 350 male and 350 female mosquitoes (50) from each village each month (caught by CDC traps) was determined by a modified cold anthrone test for fructose [33]. In some months, when the catch of mosquitoes was low, the entire catch was tested. In ASB catches, single mosquitoes were taken with forceps from opaque white trap catch-bags and placed into microtitre plates. Workers did not to look into the bag to avoid biased selection. Anthrone test reagent containing 0.15% w/v anthrone (Sigma, St Louis MO, USA) w/v in 71.7% sulfuric acid was used. Each mosquito was placed in the well of a flat-bottomed microtitre plate and wetted with 20 μl of 100% ethanol. Aliquots of 200 μl reaction solution were added to the wells and the specimens were crushed with a glass rod that was repeatedly washed with water and wiped. Crushed mosquitoes were examined after incubation for 60 min at 25 °C and sugar meals were identified by development of blue/green colour. Dye marked bait fed mosquitoes containing food dye were first identified visually with a simple magnifying glass and then re-examined under a dissection microscope. Any blue colour development was considered as positive for sugar. Wells that stayed yellow were considered negative. For a visual indicator of blue colour change see [34].

### ATSB composition, ASB composition and bait station construction

Attractive toxic sugar baits contained the active ingredient dinotefuran 0.11% (w/w), 1% (w/w) BaitStab—a product containing antibacterial and antifungal additives (Westham Innovations LTD., Tel Aviv, Israel), 77% (w/w) brown sugar, and 22% (w/w) date syrup. ASB was similar but without toxin and with food dye (Tartrazine 19140, Stern, Natanya, Israel) were provided by Westham Innovations LTD (Tel Aviv, Israel). Bait stations were constructed using a white, rectangular plastic frame with the ASB or ATSB inside with proprietary, mosquito bite and emanation-permeable, black plastic membrane cover and 100 g of the bait were inserted into the 16 cells of membrane (Westham Innovations LTD, Tel Aviv, Israel). ATSB bait stations (Additional file 2: Fig. S2) were hung (2 per house) in treated villages on May 31st and were left until the end of the project in December.

The AI is an oral toxin, a neonicotinoid [35], with low toxicity to mammals, yet is an acetylcholine agonist affecting the nervous system of insects when ingested.

### Study of the population composition

Mosquito populations in all the 14 villages were sampled monthly in 2017 from 3 months before the peak mosquito season in April and until 2 months after the peak of the mosquito season in December. Post-collection processing included mosquito identification, age grading, and *Plasmodium falciparum* sporozoite determination by ELISA. Vector population densities was determined using the following methods.

### CDC traps

In each village, trapping was at the approximate centre where houses were closer together and a near-grid pattern could be obtained for good coverage. 10 CDC UV light traps (Model 512, John W. Hock Company, Gainesville, Florida, USA) were set up outdoors at least 10.0 m apart, in each village; Their location was in a rough grid pattern next to 10 houses (with permission of the owners) about 5.0 m away from the house. Traps were set at 18:00 h and were emptied at 06:00 h. This was conducted in each village for one night per month.

### Human landing catches

The United States Environmental Protection Agency (EPA) guidelines and protocols for the use of human volunteers in HLC experiments were carefully followed [36]. Four volunteers, all male professional entomologists, participated in this study. As part of the informed consent process, the participants were fully advised of the nature and objectives of the test and the potential health risks from exposure to mosquito bites. According to EPA regulations, they were required to avoid alcohol, caffeine, and fragrance products (e.g., perfume, cologne, hairspray, lotion, etc.) during the entire test period. For the mosquito collection tests, volunteers were wearing long trousers and long-sleeved shirts as protection against mosquito bites. One leg of the trousers was rolled up to expose the skin used as the test area. The volunteers were seated motionless in chairs with the exposed leg extended while observation, counting, collecting and recording of mosquitoes was made. The distance between the outdoor volunteers was at least 5 m and indoor volunteers were located in separate homes. Both indoor and outdoor volunteer locations were switched every 2 h from 18:00 to 06:00 h to eliminate positional bias. Collections were made in two bedrooms of two separate homes per village per month. Mosquitoes for sampling were collected with an entomological hand-vac (Mosquito and Sand fly aspirator model 419; John W. Hock Company, Gainesville, Florida USA) which was used to aspirate landing/biting mosquitoes off of the human volunteer.

### Malaise traps

Ten standard 6 m malaise traps (Model 3012, John W. Hock Company, Gainesville, Florida, USA) were set up to trap flying adult mosquitoes. These were set at least 10 m apart and further from the centre of the village where houses were more crowded together. Because these traps are larger than CDC, they were positioned 10 m from the houses. Traps were set at 18:00 h and were emptied at 06:00 h.

### Pyrethrum spray catches

Pyrethrum spray catches indoor collections in 10 bedrooms of 10 separate homes per village once per month were conducted according to established protocols [37] to determine the density of resting mosquitoes in homes.

### Age determination

Random samples of 200 females (whenever seasonally possible) collected by each trapping method, were analyzed and the physiological age (number of past ovipositions) was determined by dissection and examination of ovaries in a drop of PBS under a dissecting microscope 10× to 100×, to expose and count the dilatations in ovarioles [38]. Females were then classified as having undergone either less than 3 gonotrophic cycles or 3 or more cycles.

### ELISA testing

A *P. falciparum* “sandwich” ELISA was used to test female mosquitoes for sporozoites according to standard protocols [39]. A subsample of 150 randomly selected female *An. gambiae* s.l. (whenever seasonally possible) collected by each mosquito collection method per village per month, to be processed by ELISA. If the total monthly catch per collection method was less than 150 specimens, all available material was processed by ELISA.

### Determination of entomological inoculation rate

The entomological inoculation rate (EIR) was calculated for mosquitoes caught landing/biting the human volunteers (HLCs). The EIR which is a measure of exposure to infected mosquitoes, is defined as the product of the mosquito biting rate and the sporozoite rate [40]. In this case, the mean daily entomological inoculation rate was calculated by multiplying the overall monthly sporozoite rate determined by ELISA (for all females tested per village from HLC catches) times the mean daily biting pressure from control or treated villages. The biting pressure is defined as the number of biting females per person per night. The monthly inoculation rate was calculated by multiplying the mean daily EIR by 30.

### Determination of sporozoite infection

A “sandwich” ELISA was used to test mosquitoes for sporozoites according to the protocol detailed in [39].

### Statistical analysis

For the 2-day experiment, a generalized mixed linear model for a Poisson distributed outcome was used. Because the data exhibited over-dispersion, a negative binomial analysis was used. The fixed effects were month, treatment (Anthrone vs. ASB), and the interaction of month and treatment. Treatment is a repeated measure, and a random error term of village nested in month was used to provide an error term for the repeated measure. A heterogeneous compound symmetric covariance matrix was used to represent the correlated data structure. Model mean percent, standard error, and 95% CI of the difference between means as well as P-value for a comparison between treatments at each month is presented. Population density had Poisson distribution. Over-dispersion was evident; therefore, a generalized mixed linear model for a negative binomial distribution to analyze the data for each of the three trap types: CDC, Malaise, and PSC was used separately. The model included fixed effects for month (April–December), treatment (control and experimental: a repeated measure over months), and the interaction of month and treatment. A random error term of villages nested within treatment was used for the error term for treatment. A compound symmetric covariance matrix was used to represent the correlated data structure. Model means and standard errors as well as 95% confidence intervals (CIs) for mean differences are presented for the interaction. P-values are also presented for planned comparisons between treatments at each month. Human landing catches also had a Poisson distribution with over-dispersion; the same analysis plan was used as described above for the traps. The gonadal age and sporozoite infection rate data both had binomial distributions, therefore, a generalized linear mixed model was used to analyze these data. The same model as for the trap data described above was used for both. Model mean and standard error as well as 95% CIs for mean differences are presented for the interactions. P-values are also presented for planned comparisons between treatments at each month. A general linear model analysis for repeated measures was performed for the monthly EIR rates for indoor and outdoor data separately. The outcome was the EIR; predictors were group (control vs. treatment), month (April–December) and the interaction of group and month. A random effect of village nested within group was included to provide an error term for the repeated measures over month. A compound symmetric covariance matrix was used to represent the correlated structure of the data. Model means and standard errors were reported as well as P-values for comparisons between the groups at each month. The percent reduction in EIR due to the treatment is also included. The two-tailed alpha level was used to determine statistical significance. SAS 9.4 (SAS Institute Inc; Cary NC) was used for all analyses.

## Results

### Feeding of malaria vectors on sugar from natural sources compared to feeding from attractive bait stations (ASB)

**Fig. 1:**
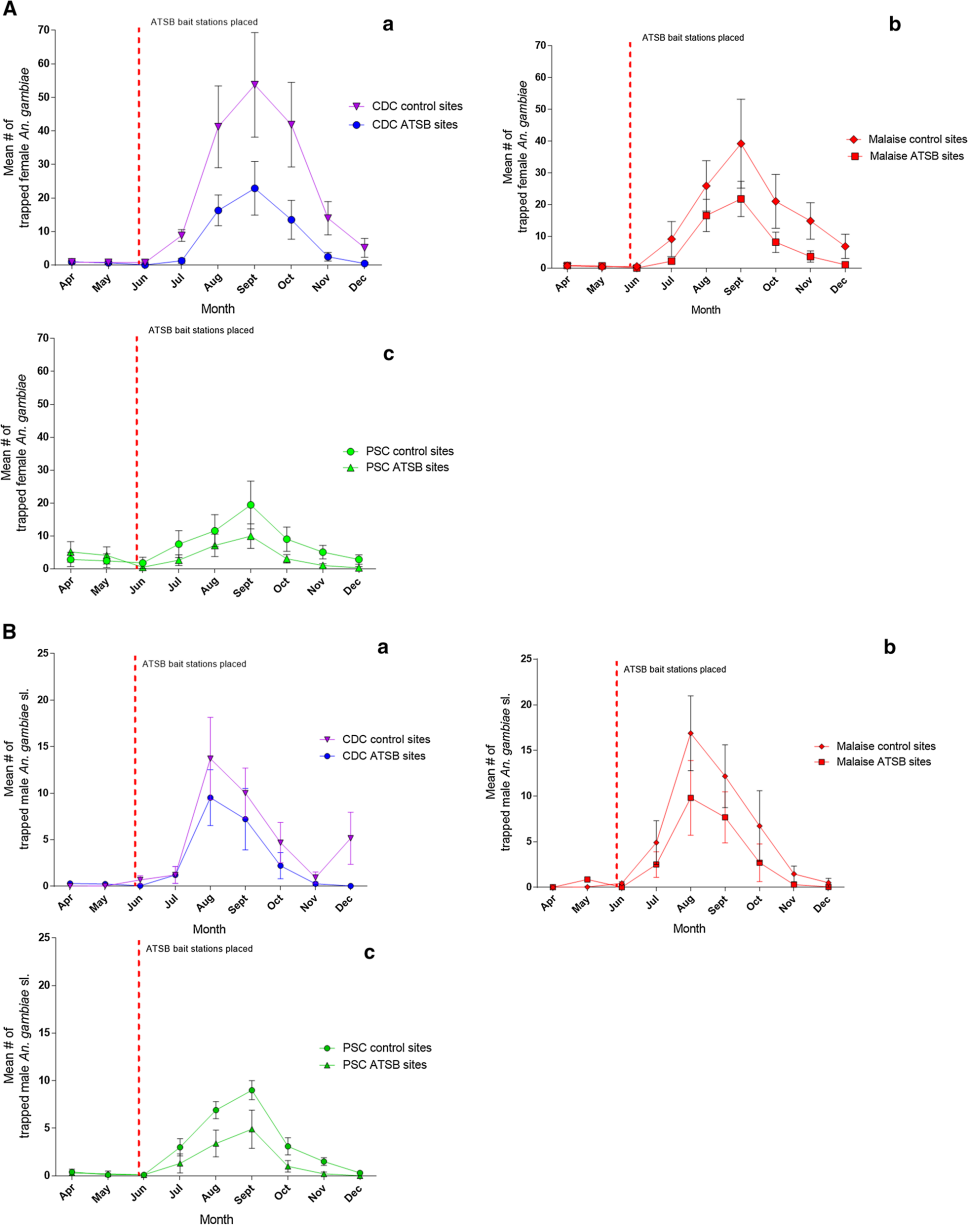
**A** Comparison of the mean number of female *Anopheles gambiae* s.l. (± SE) collected per trap per village from ATSB treated and control villages. The pre-treatment monitoring period was from April until ATSB treatment on May 31st Control and experimental sites were monitored from June to December. **a** catch of CDC traps; **b** catch of Malaise traps; **c** PSCs. **B** Comparison of the mean number of male *Anopheles gambiae* s.l. (± SE) collected per trap per village from ATSB treated and from control villages. The pre-treatment monitoring period was from April until ATSB treatment in the 31 of May; All sites were monitored from June to December. **a** Catch of CDC traps; **b** Catch of Malaise traps; **c** PSC catches

In the 2-day baseline experiment in 2016, the mean number of females from control villages that fed on natural sugar (i.e. anthrone positive) reached a maximum of 44.9% of the catch (22.43 ± 1.70 SE per trap) in August, while males maximal sugar feeding of 45.1% was observed in September (22.57 ± 1.46 SE per trap) (Fig. 1A, Tables 2, 3). Presentation of ASB bait stations lowered the feeding on natural sources and the percentage of anthrone positive mosquitoes was exceeded by the number of ASB dye-marked mosquitoes (Fig. 1B, Table 2). In the control site the mean number of females feeding on ASB reached a maximum of 35.0% of the catch (17.57 ± 2.37 SE per trap) in September and they were 17.0% 10.86 ± 1.79 SE per trap in August. The number of males feeding on ASB reached a maximum of 39.9% of the catch (15.43 ± 1.53 SE per trap) in November while those that fed on natural sugar sources were only 22.3% in August (8.00 ± 1.21 SE; Tables 2, 3).

**Table 2:** Baseline mean (± SE) of male and female *An. gambiae* that had natural sugar in the gut before presentation of ASB (Day 1)

Month	Day 1—Anthrone positive (Natural sugar-fed)				
	Female			Male	
	Mean^a^ ± SE	% of catch	Month	Mean^a^ ± SE	% of catch
Apr N = 85	2.57 ± 1.08	21.10	Apr N = 2	0.28 ± 0.18	0.00
May N = 65	1.17 ± 0.77	18.90	May N = 1	0.14 ± 0.14	0.00
Jun N = 75	3.42 ± 1.19	32.00	Jun N = 2	0.28 ± 0.28	0.00
Jul N = 350	17.71 ± 3.02	39.70	Jul N = 140	20.00 ± 2.43	44.00
Aug N = 350	22.43 ± 1.70	44.90	Aug N = 145	20.86 ± 1.22	41.80
Sept N = 350	20.57 ± 2.26	41.10	Sept N = 160	22.57 ± 1.46	45.10
Oct N = 350	18.86 ± 1.92	37.70	Oct N = 125	17.86 ± 1.71	41.80
Nov N = 300	13.43 ± 1.64	31.30	Nov N = 15	2.28 ± 1.07	28.10
Dec N = 250	8.14 ± 2.72	22.70	Dec N = 1	0.14 ± 0.14	0.00

N = Total pooled sample size from all villages

^a^Mean positive per village

**Table 3:** Comparison of the means of males and females feeding on natural sugar versus those feeding on ASB per village (± SE with 95% CI of the difference between means)

Month	Day 2—Anthrone positive (Natural sugar-fed) vs. ASB positive												
	Female anth	Female ASB						Male anth	Male ASB				
	Mean^a^ ± SE	Mean ± SE	Diff	95% CI^b^		P value	Month	Mean^a^ ± SE	Mean ± SE	Diff	95% CI^b^		P value
Apr N = 88	0.71 ± 0.29	5.86 ± 1.65	5.15	7.42	2.88	< 0.0001	Apr N = 2	0.00 ± 0.00	0.14 ± 0.14	0.14	1.32	0.00	0.5010
May N = 60	0.43 ± 0.20	3.71 ± 1.17	3.28	5.55	1.01	0.0010	May N = 1	0.00 ± 0.00	0.00 ± 0.00	0.00	1.18	0.00	0.9720
Jun N = 80	0.57 ± 0.30	4.29 ± 1.87	3.72	5.99	1.45	0.0010	Jun N = 3	0.14 ± 0.14	0.14 ± 0.14	0.00	1.18	0.00	0.9940
Jul N = 320	7.57 ± 1.54	16.14 ± 2.23	8.57	10.84	6.30	0.0050	Jul N = 310	5.86 ± 0.63	15.29 ± 1.19	9.43	10.61	8.25	< 0.0001
Aug N = 350	10.86 ± 1.79	13.86 ± 1.75	3.00	5.27	0.73	0.0034	Aug N = 350	8.00 ± 1.21	15.43 ± 1.53	7.43	8.61	6.25	< 0.0001
Sept N = 350	6.71 ± 1.04	17.57 ± 2.37	10.86	13.13	8.59	< 0.0001	Sept N = 350	5.71 ± 0.61	15.71 ± 1.34	10.00	11.18	8.82	< 0.0001
Oct N = 350	6.71 ± 1.13	15.14 ± 1.99	8.43	10.70	6.16	0.0030	Oct N = 300	6.00 ± 0.93	13.86 ± 1.03	7.86	9.04	6.68	< 0.0001
Nov N = 300	2.71 ± 0.68	16.00 ± 2.17	13.29	15.56	11.02	< 0.0001	Nov N = 60	0.29 ± 0.18	3.29 ± 1.04	3.00	4.18	1.82	0.0020
Dec N = 250	1.71 ± 0.52	11.57 ± 1.38	9.86	12.13	7.59	< 0.0001	Dec N = 5	0.14 ± 0.14	0.29 ± 0.29	0.15	1.33	0.00	0.9980

Catches of the first night post-presentation of ASB (Day 2)

N = Total pooled sample size from all villages

*Diff* difference between means

^a^Mean positive per village

^b^Column 1 = upper limit, column 2 = lower limit

### Population density

The mean population density of male and female mosquitoes in the control and the experimental villages was not significantly different from April to the end of May 2017, which was the pre-treatment monitoring period, (Figs. 1a, b as well as Tables 4A, B and 5). After presenting the ATSB baits, the highest mean catch of CDC light traps, obtained in September, at the control sites was 53.76 ± 15.60 SE females, and it dropped by 53.4% after ATSB treatment to 22.91 ± 8.00 SE. The maximum mean number of males, in August, was 13.71 ± 4.43 SE at the control and at the experimental site the mean was relatively reduced by 57.4% to 9.52 ± 3.00 SE. The greatest reductions, 94.7% of females and 94.0% of males by ATSB treatment, occurred in July. In ATSB treated villages the reduction in malaise trap catches was also consistently high with the maximum mean number of females, reached in September at both sites, lowered to 21.83 ± 5.55 SE compared to 39.20 ± 14.00 SE at the control, a 43.3% reduction. Males reached their maximum in August and was 9.80 ± 4.09 SE compared to 16.88 ± 4.10 SE at the control sites, a 41.9% reduction. The greatest reductions by ATSB as expressed in catches of malaise traps mean catch was 84.0% smaller in June (females) and no males (100.0% reduction) were caught in December. PSCs caught maximal number a mean of 9.99 ± 3.73 SE females in September compared to a mean of 19.46 ± 7.25 SE at the control sites, a 48.7% reduction. The maximum mean number of males 4.90 ± 2.00 SE was reached in September compared to a mean of 9.00 ± 1.00 SE at the control sites, a 45.6% reduction. The greatest reductions in female numbers, in PSC catches was in December and males completely disappeared (100.0% reduction) males (Table 4A, B). Figure 3 shows reductions in female *An. gambiae* after treatment in May measured by HLC. Indoors, the maximum number of females was caught in September with a mean of 85.36 ± 3.49 SE at the control sites which was reduced to 68.50 ± 3.13 SE at the treated sites, a reduction of 19.8%. Outdoors, the maximum number of females at the control sites was a mean of 92.21 ± 3.6 SE that were caught in September Their number declined to a mean of 67.93 ± 3.12 SE at the treated sites, a reduction of 26.3%. The greatest indoor reductions of 67.7% occurred in December and outdoors the decrease of 80.7% occurred in June (Table 5).

**Fig. 2:**
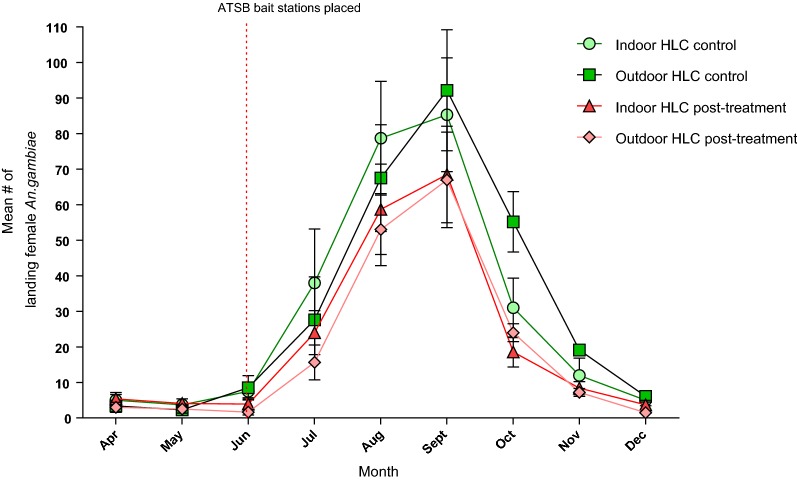
Landing/biting of *Anopheles gambiae* females in HLC observations after placement of ATSB bait stations. Data collected indoor and outdoor from ATSB treated villages, and from control villages, were grouped and the mean number of landing/biting per volunteer per night was calculated for each month. **a** Indoor, **b** outdoor

**Table 4:** **A** Percentage reduction of female *Anopheles gambiae* s.l. per trap per village observed comparing ATSB treated sites and control sites; 95% CIs of the mean difference are shown in Fig. 1A. **B** Reduction of *An. gambiae* s.l. male catches per trap in ATSB treated sites and control sites with 95% CIs of the mean difference is shown in Fig. 1A

	CDC ATSB sites vs CDC control sites					Malaise ATSB sites vs Malaise control sites					PSC ATSB sites vs PSC control sites				
Month	Diff.	95% CI UL	95% CI LL	% reduct	P value	Diff.	95% CI UL	95% CI LL	% reduct	P value	Diff.	95% CI UL	95% CI LL	% reduct	P value
A^a^															
Apr	0.06	0.41	0.00	N/A	0.8860	0.05	1.75	0.00	N/A	0.8300	2.30	6.35	0.00	N/A	0.1830
May	0.11	0.58	0.00	N/A	0.9070	0.33	1.47	0.00	N/A	0.4170	1.63	6.02	0.00	N/A	0.2620
Jun	0.72	1.19	0.00	94.74	0.0140	0.49	1.29	0.00	83.05	0.0184	1.31	5.96	0.00	71.20	0.0780
Jul	7.54	17.01	0.00	85.39	0.0010	6.95	14.75	0.00	75.63	0.0030	4.85	9.50	0.20	64.15	0.0180
Aug	24.95	34.42	15.48	60.47	0.0080	9.30	17.10	1.50	35.89	0.0171	4.44	9.09	0.00	38.18	0.0220
Sept	30.85	40.34	21.38	57.38	0.0090	17.38	25.18	9.58	44.33	0.0460	9.47	14.12	4.82	48.66	0.0400
Oct	28.36	37.83	18.89	67.75	0.0010	12.88	20.68	5.08	61.13	0.0060	5.95	10.60	1.30	65.67	0.0100
Nov	11.48	20.95	2.01	82.18	0.0010	11.20	19.00	3.40	75.22	0.0010	4.07	8.72	0.00	79.18	0.0030
Dec	4.7	14.17	0.00	91.09	0.0010	5.79	13.59	0.00	84.03	0.0010	2.53	7.18	0.00	87.24	0.0060
B^b^															
Apr	0.29	2.54	− 3.12	N/A	0.9200	0.00	3.20	0.00	N/A	0.8300	0.10	1.29	0.00	N/A	0.1830
May	0.21	2.62	− 3.04	N/A	0.5580	0.83	2.37	0.00	N/A	0.4170	0.10	1.09	0.00	N/A	0.2620
Jun	0.63	3.46	− 2.20	94.03	0.0076	0.44	3.64	0.00	100.00	0.1840	0.00	1.19	0.00	0.00	0.0480
Jul	0.01	2.84	− 2.82	0.83	0.0010	2.40	5.60	0.00	48.98	0.0030	1.70	2.89	0.51	56.67	0.0180
Aug	4.19	7.02	1.36	30.56	0.0110	7.08	10.28	3.88	41.94	0.1710	3.50	4.69	2.31	50.72	0.0220
Sept	2.81	5.64	− 0.02	28.07	0.0190	4.50	7.70	1.30	36.98	0.0760	4.10	5.29	2.91	45.56	0.0400
Oct	2.48	5.31	− 0.35	52.99	0.0080	4.02	7.22	0.82	59.82	0.0060	2.10	3.29	0.91	67.74	0.0100
Nov	0.67	3.50	− 2.16	72.83	0.0015	1.18	4.39	0.00	80.54	0.0001	1.30	2.49	0.11	86.67	0.0030
Dec	5.14	7.97	2.30	99.73	0.0039	0.46	3.66	0.00	92.00	0.0010	0.30	1.49	0.00	100.00	0.0060

Horizontal line—ATSB treatment

*N/A* not applicable, pre-treatment monitoring period, *Diff.* difference between means, *UL* upper limit, *LL* lower limit

^a^P values of the comparison between mean numbers of females caught per trap per village in Fig. 1A are also shown

^b^P values of the comparison between mean numbers of males caught per trap in Fig. 1A are also shown

**Table 5:** Reduction of indoor and outdoor landing/biting of female *An. gambiae* s.l. per volunteer per night, from control sites compared to ATSB treated sites

Month	Indoor					Outdoor				
	Diff.	95% CI UL	95% CI LL	% reduct	P value	Diff.	95% CI UL	95% CI LL	% reduct	P value
Apr	0.50	8.39	− 9.39	N/A	N/A	0.43	10.63	− 9.77	N/A	N/A
May	0.43	8.46	− 9.32	N/A	N/A	− 0.07	10.13	− 10.27	N/A	N/A
Jun	3.64	12.54	− 5.25	48.11	0.014	6.20	16.40	4.00	80.67	0.006
Jul	8.07	16.96	0.82	37.08	0.046	11.21	21.41	1.01	43.41	0.043
Aug	7.29	16.18	1.61	25.32	0.042	6.22	16.42	3.98	20.85	0.075
Sept	2.79	11.68	0.11	19.75	0.046	6.19	16.38	4.01	26.34	0.064
Oct	13.36	22.25	4.46	42.34	0.018	15.36	25.56	5.16	55.30	0.011
Nov	7.93	16.82	0.96	65.29	0.033	5.32	15.52	0.88	61.94	0.034
Dec	4.36	13.25	0.54	68.68	0.025	1.82	12.02	0.38	74.42	0.043

(Differences are significant if P < 0.05)

Horizontal line—ATSB treatment

*N/A* not applicable, pre-treatment monitoring period, *Diff.* difference in means

**Fig. 3:**
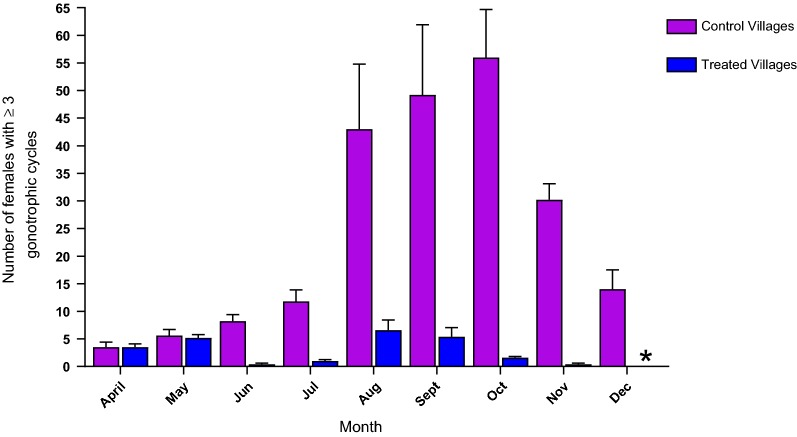
The mean number per village of *Anopheles gambiae* s.l. females that passed ≥ 3 egg laying cycles in control villages and after treatment with ATSB. *Indicates absence of mosquitoes

### Age determination

After ATSB treatment, reductions in the mean number of older females that passed three or more than egg laying cycles were observed in all months. Notably, the highest mean number of 56.00 ± 8.69 SE older females was recorded in October in the control villages while in parallel ATSB treated villages their mean number was 1.6 ± 0.21 SE and such females were completely absent in December (Table 6).

**Table 6:** Comparison of the number of female *An. gambiae* s.l. that passed ≥ 3 egg laying cycles from control sites and ATSB treated sites after the pre-treatment monitoring period

ATSB (N)	ATSB sites	Control sites						
	Mean ± SE	Control (N)	Mean ± SE	Diff.	95% CI UL	95% CI LL	% reduct	P value
Apr N = 160	3.50 ± 0.60	Apr N = 160	3.50 ± 0.90	0.00	7.21	− 7.21	N/A	0.6960
May N = 158	5.20 ± 0.60	May N = 158	5.60 ± 1.10	0.40	7.61	− 6.81	N/A	0.4000
Jun N = 220	0.40 ± 0.21	Jun N = 220	8.20 ± 1.18	7.80	15.01	0.59	95.12	0.1480
Jul N = 600	1.00 ± 0.27	Jul N = 600	11.80 ± 2.13	10.80	18.01	3.59	91.53	0.0110
Aug N = 600	6.60 ± 1.80	Aug N = 600	43.00 ± 11.78	36.40	43.61	29.19	84.65	< 0.0001
Sept N = 600	5.40 ± 1.66	Sept N = 600	49.20 ± 12.70	43.80	51.01	36.59	89.02	< 0.0001
Oct N = 600	1.60 ± 0.21	Oct N = 600	56.00 ± 8.69	54.40	61.61	47.19	97.14	< 0.0001
Nov N = 370	0.40 ± 0.21	Nov N = 370	30.20 ± 2.91	29.80	37.01	22.59	98.68	< 0.0001
Dec N = 135	0.00 ± 0.00	Dec N = 135	14.00 ± 3.48	14.00	21.21	6.79	100.00	0.9630

*N/A* not applicable, *Diff*. difference between means

N = Total number dissected from CDC, malaise and PSC catches

Horizontal line—ATSB treatment

### Entomological inoculation rate (EIR) and sporozoite presence in mosquitoes caught on human volunteers

Following ATSB treatment, there were high reductions of 77.76% to 100% indoors and 84.95% to 100% outdoors in monthly mean of EIRs (Table 7A, B). In September, the EIR at the control houses indoors was very high at 70.71 while inside the ATSB treated houses it was only 10.71 females. In the same month, outdoors at the control site, the EIR was 57.93 while at the ATSB treated site it was 6.45 females (so many times smaller).

**Table 7:** Monthly means of biting pressure, sporozoite infection in females (determined by ELISA), and the frequency of entomological inoculation (EIR)

A	Indoor treated sites					Indoor control sites					Monthly EIR reduction (%)	P-value
Month	Monthly Ave BP	Ave tested^b^	Ave # infected	Infection rate (IR)	Monthly EIR	Monthly Ave BP	Ave tested^b^	Ave # infected	Infection rate (IR)	Monthly EIR		
Apr	162.86	10.86	0.00	0.0000	0.00	147.86	9.86	0.00	0.0000	0.00	N/A	N/A
May	124.29	8.29	0.00	0.0000	0.00	111.43	7.43	0.00	0.0000	0.00	N/A	N/A
Jun	117.86	7.86	0.00	0.0000	0.00	227.14	14.86	0.29	0.0200	4.37	100.00	0.3085
Jul	720.00	48.00	0.29	0.0100	4.29	962.14	64.14	1.29	0.0200	19.29	77.76	0.0880
Aug	1527.86	101.57	0.29	0.0030	4.30	1746.43	116.86	3.00	0.0300	44.83	90.41	< 0.001
Sept	1714.29	114.29	0.71	0.0100	10.71	1797.86	119.86	4.71	0.0400	70.71	84.85	< 0.001
Oct	548.57	36.57	0.43	0.0100	6.43	949.29	63.29	3.14	0.0500	47.14	86.36	< 0.001
Nov	126.43	8.43	0.00	0.0000	0.00	364.29	24.29	0.29	0.0100	4.29	100.00	0.9999
Dec	47.14	3.14	0.00	0.0000	0.00	177.86	11.86	0.00	0.0000	0.00	N/A	N/A

B	Outdoor treated sites					Outdoor control sites					Monthly EIR reduction (%)	P-value
Month	Monthly Ave BP	Ave Tested^b^	Ave # Infected	Infection Rate (IR)	Monthly EIR	Monthly Ave BP	Ave Tested^b^	Ave # Infected	Infection Rate (IR)	Monthly EIR		
Apr	90.00	6.00	0.00	0.0000	0.00	102.86	6.86	0.00	0.0000	0.00	N/A	N/A
May	72.86	4.86	0.00	0.0000	0.00	70.71	4.71	0.00	0.0000	0.00	N/A	N/A
Jun	69.00	4.60	0.00	0.0000	0.00	255.00	17.00	0.14	0.0100	2.14	100.00	0.9279
Jul	469.29	31.29	0.00	0.0000	0.00	805.71	55.29	1.14	0.0200	16.66	100.00	0.0050
Aug	1407.86	102.00	0.43	0.0040	6.45	1534.29	93.86	2.86	0.0300	42.86	84.95	0.0010
Sept	1673.57	114.86	0.43	0.0040	6.45	1729.29	111.43	3.86	0.0300	57.93	88.87	< 0.001
Oct	370.71	24.71	0.14	0.0100	2.14	831.43	55.14	3.29	0.0600	49.54	95.68	< 0.001
Nov	127.50	8.50	0.00	0.0000	0.00	287.14	19.14	0.14	0.0100	2.14	100.00	0.9279
Dec	37.50	2.50	0.00	0.0000	0.00	92.14	6.14	0.00	0.0000	0.00	N/A	N/A

Measurements were made using human landing catches. A) Indoor B) Outdoor

*N/A* Not applicable, no reduction

^a^BP = (#bites/person/night)

^b^Average out of a maximum sample size of 150/village

IR = # Infected/# tested

Horizontal line = ATSB treatment

Indoors, there were decreases in monthly *P. falciparum* sporozoite rates (based on ELISA testing). The mean rate of sporozoite positive mosquitoes indoors in the control villages ranged from 0.84 to 3.8% while the mean rate of sporozoite positive mosquitoes indoors in the ATSB treatment villages ranged from 0.38 to 0.61% (Table 7A). In these villages, no sporozoite positive mosquitoes were detected early in the transmission season (June) or at the end of the transmission season (November and December).

Outdoors, the mean rate of sporozoite positive mosquitoes in control villages ranged from 0.59 to 3.5% with no positive mosquitoes in April, May or December. In treated villages, the outdoor sporozoite infection rate ranged from 0.28 to 0.57%, but no sporozoites were detected from April to July and again in November and December (Table 7A, B).

### Sporozoite rate in mosquitoes caught using all trapping methods

**Fig. 4:**
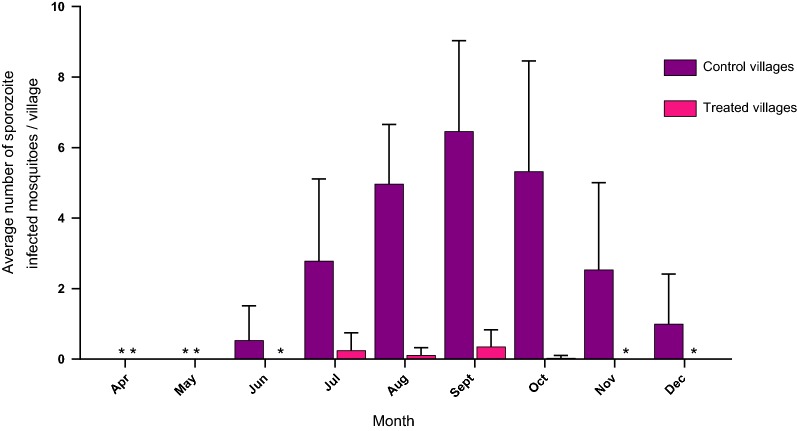
Mean number per village and month (± SE) of female *Anopheles gambiae* s.l., collected from all trapping methods and found infected with sporozoites in control villages compared to treated villages

Figure 4 shows the comparison of the mean number of sporozoite positive female mosquitoes from all trapping methods, from control sites compared to treated sites is shown in Fig. 4. Mosquitoes sampled by all trapping methods (CDCs, Malaise, PSCs, and HLCs) and tested by ELISA for sporozoites, were as expected, negative for the first 2 months both in control and treated sites (April and May). From July to October, when catches were highest, sporozoite positive females were reduced by 84.97% to 95.74% respectively at the ATSB site compared to the control site (Table 8).

**Table 8:** Monthly comparison of the mean number of sporozoite infected female *An. gambiae* s.l. at the ATSB treated sites versus the control sites

Month	ATSB sites	Month	Control sites	Diff.	95% CI UL	95% CI LL	% reduct	P value
ATSB (N)	Mean ± SE	Control (N)	Mean ± SE					
Apr N = 120	0.00 ± 0.00	Apr N = 120	0.00 ± 0.00	0.00	N/A	N/A	N/A	N/A
May N = 120	0.00 ± 0.00	May N = 120	0.00 ± 0.00	0.00	N/A	N/A	N/A	N/A
Jun N = 220	0.00 ± 0.00	Jun N = 220	0.14 ± 0.49	0.54	3.35	− 2.28	100.00	0.9996
Jul N = 600	0.00 ± 0.25	Jul N = 600	1.14 ± 1.16	2.54	5.35	− 0.28	100.00	0.1035
Aug N = 600	0.43 ± 0.11	Aug N = 600	2.86 ± 0.85	4.86	7.67	2.05	84.97	< 0.0001
Sept N = 600	0.43 ± 0.24	Sept N = 600	3.86 ± 1.28	6.11	8.92	3.30	88.86	< 0.0001
Oct N = 600	0.14 ± 0.04	Oct N = 600	3.29 ± 1.57	5.29	8.10	2.48	95.74	< 0.0001
Nov N = 360	0.00 ± 0.00	Nov N = 360	0.14 ± 1.24	2.54	5.35	− 0.28	100.00	0.1035
Dec N = 120	0.00 ± 0.00	Dec N = 120	0.00 ± 0.71	1.00	3.81	− 1.81	N/A	0.9646

*N/A* Not applicable, no reduction, *Diff.* difference between treatment and control means

Horizontal line—ATSB treatment

N = Total number tested from CDC, malaise, PSC and HLC catches

## Discussion

This field trial in southern Mali demonstrated that the use of ATSB had significant diminishing effects on the presence of malaria vectors and consequently on malaria parasite transmission [7, 9]. The effect was manifested by reductions in vector density and biting pressure and specifically by near elimination of ‘old’ females that passed at least three egg laying cycles and have a high potential as malaria vectors. Both the sporozoite prevalence, and EIR were also lowered.

The current study used an ATSB system with the AI dinotefuran, a neonicotinoid agonist of the acetylcholine receptor that affects the nervous system [35]. It works when ingested and thereafter has a systemic action with low toxicity mammals [40] which better for the environment.

Estimation of ATSB for mosquito control depended on the earlier observations that sugar meals from natural sources are staple diet for female and male mosquitoes [19, 20]. In this case, a primary test was to examine the frequency of sugar feeding from natural sources by the local malaria vectors to assess the potential of using ATSB. It was observed that similar proportions of males and females, ranging from ~ 20 to ~ 50% of the caught mosquitoes, depending on the season, feed on natural sugar sources (males were scarce in the driest months, therefore, their sugar feeding was not recorded in Fig. 1A). After ASB bait stations were hung, the results show that a high proportion of the sugar questing mosquitoes preferred to feed on ASB instead of natural sugar sources (Fig. 1B). The preference of ASB prevailed regardless of season and thus persisted even if there is a seasonal variation in natural sugar sources. The advantage of ASB also shows that the chosen location of the baits in the villages was successful. This demonstrates that ASB, and presumably also ATSB, compete favourably with locally available sugar sources in Mali. Moreover, these results imply that the use of ATSB, a toxin-containing ASB in similar environmental conditions can cause persisting decrease of the malaria vector populations.

Indeed, the use of ATSB bait stations outdoors in seven experimental villages was associated with high continuous reductions in population densities of female and male *An. gambiae* s.l. compared to populations exposed to ASB in seven control villages. These results were repeatedly confirmed in mosquito samples that were captured by different concurrently employed trapping methods. Similar results showing high proportions of ASB marked mosquitoes, as well as ATSB mosquito reductions were obtained in previous small studies in Israel and elsewhere in Mali [21, 27, 41–43]. All types of trapping methods showed some reduced effect of ATSB in the wetter months (August to October).; CDC trap catches were reduced by ~ 15 to 20% while malaise trap and PSC catches were reduced 20 to 30% during the same months (Figs. 1 and 2a–c). The reduced impacts as population densities increase during the rainy season are likely because the experimental area is on the floodplains of the River Niger and apparently this causes a massive influx of young mosquitoes from rice fields to rice growing villages. It is important to note that in previous studies indicate bait stations aged under field conditions maintain their attraction and mosquito killing activity for 6 months (unpublished data) and so reduced activity of the ATSB unit would not be the cause of reduced ATSB impact. Population reductions in October to December were greater than during the peak months of transmission (July/August), likely because of the observed drying up of neighbouring breeding sites that limited the emergence of new mosquitoes. Another decrease in the presence of mosquitoes occurred indoors and was expressed in yields of HLC and PSC methods. This decrease followed and was caused by the location of bait station outdoors.

Mosquito vectors become infected by feeding on human blood with malaria parasites and they can transmit sporozoites to humans after a 10 to 18 day cycle of growth [44]. Hence only mosquitoes older than 10 days that may carry infective malaria sporozoites can be effective vectors. In previous studies such mosquitoes were defined by evidence that they passed at least three egg-laying cycles of 4 days each [44]. As shown in this field study, following the ATSB outdoor treatment the diminishing of the older female group is much greater than that of the general female population. It is hypothesized that this occurs since in the continuous ATSB treatment, the exposure of mosquitoes to the toxin and mosquito mortality increases with age and it is highest in the group of older females, the vectors of malaria. In previous studies the reductions of > 85% during August to October, when the highest proportions of older females were expected, shows how ATSB distinctly reduces the group of mosquitoes that reach this age and has the direct cumulative effect on older, more dangerous, females that have a higher chance of feeding on toxic sugar. These results are in agreement with previous studies [28, 45].

The reduction of females infected with sporozoites is an important entomological test of an intervention against malaria and it is of particular interest because it may directly affect human health. The results in this study show how the use of ATSB bait stations outdoors can significantly reduce the number of females that are infective and capable of transmitting sporozoites to humans by bite. After ATSB treatment both indoors and outdoors, the incidence of sporozoite infection in *An. gambiae* females never exceeded 1%, and no infection could be detected in the drier months. According to the size of samples mosquito populations are small in these months and this may be the period in which ATSB has the best chance, combined with additional interventions, such as LLINs, to block the transmission cycle of malaria.

The use of ATSB outdoor bait stations was also associated with major reductions in EIRs. In a previous study [46], use of insecticide (permethrin) treated nets (ITS) alone reduced EIRs in Tanzania, Solomon Islands, and Kenya to between 45 and 90%. In the current study, the impact of adding outdoor ATSB bait stations to indoor deltamethrin LLINs compared to indoor deltamethrin LLINs alone, reduced the monthly EIRs indoors by > 80% when transmission was high and by 100% when transmission was low (Table 7A). Outdoors, where the baits stations were placed, EIR reductions were at least as high as 92% and also reached 100% in low transmission months (Table 7B). Moreover, there was no detectable EIR transmission in the ATSB treatment villages during the very beginning (June, July) or at the end (November, December) of the seasonal transmission period (Table 7A and B). In areas of low transmission, where the annual EIRs are < 1 to 2, lowering of the EIR by the proportion achieved in the current study could, on its own be a promising step towards reducing transmission [47]. The reduction in EIRs were greater outdoors than indoors (Table 7A and B). This is not surprising as bait stations were hung outdoors, however, Qualls and colleagues [29] were able to demonstrate significant mosquito PSC reductions by placing bait stations indoors which could be tried alongside the outdoor ones once indoor models become more developed.

Apart from larval source management (LSM) which is not widely used to control malaria [48], current vector control tools are focused indoors. ATSB controls both indoor and outdoor populations, and this becomes even more important in the drive towards malaria elimination as reports of outdoor biting, now known as “residual transmission” [7, 49], is a new recognized set of behaviours exhibited by a mosquito which renders traditional control methods such as IRS and LLINs ineffective. The WHO in its updated malaria terminology guide book, now further recognizes residual transmission as continuing transmission following the enactment of a widely effective malaria programme. ATSB can also be useful in insecticide resistance management and IVM programs as it can use active ingredients with different modes of action because of the direct uptake during feeding.

There are some limitations to this study. First, there was a high degree of variability in trap catch numbers depending on season, and while ATSB had a greater effect on lowering EIR and sporozoite rate in the dry season when mosquito numbers were already low, it was paradoxically difficult to catch enough mosquitoes during this season to demonstrate a major impact on transmission. Second, the toxic sugar baits need to be tested for a second field season so that the results can become predictive and transferable to parts of Africa outside of Mali. Last, we are preparing a paper furthering research that studies the effect of ATSB on organisms outside of mosquitoes or so called “non-targets” such as bees and butterflies. So far, we have found the effect is predominantly on Diptera and the effects on Bees and Butterflies (Lepidoptera) can be mitigated by proper placement of the baits or application of the spray.

## Conclusion

The current large field trial in Mali shows that ATSB is competitive with local sugar sources and its use has significant impacts on population density, age groups proportions, malaria infection incidence in mosquitoes and EIR. It also demonstrates that the use of ATSB bait stations could virtually obstruct transmission in the dry season and could be an effective new class of tool to combat malaria. In order to achieve greater impact, further studies should explore the benefits of using the baits both indoors and outdoors in a range of geographical settings.

## Supplementary information

**Additional file 1: Fig. S1.** Map of the positions of the 14 study villages and their relation to the Niger River. Generated by Google Maps Professional.

**Additional file 2: Fig. S2.** A schematic presentation of bait station with a magnification of the permeable, black plastic membrane penetrable enough for mosquitoes to feed. B) Photo of the bait station hung on the outer wall of a house.

## Data Availability

All data generated and analysed during this study are included in this published article.

## Abbreviations

ASB: Attractive sugar bait
ATSB: Attractive toxic sugar baits
CDC: Center for Disease Control
CI: Confidence interval
EIR: Entomological inoculation rate
EPA: Environmental Protection Agency
HLC: Human landing catch
LLINS: Long-lasting insecticidal nets
PSC: Pyrethrum spray catch
WHO: World Health Organization
