# Supplementary material for: Large-scale field trial of attractive toxic sugar baits (ATSB) for the control of malaria vector mosquitoes in Mali, West Africa
Source: Malar J. 2020 Feb 14;19:72. doi: 10.1186/s12936-020-3132-0 (PMC7023716; doi:10.1186/s12936-020-3132-0)
Supplement: Supplementary file 1 — Additional file 1: Fig. S1. Map of the positions of the 14 study villages and their relation to the Niger River. Generated by Google Maps Professional. [file 12936_2020_3132_MOESM1_ESM.docx]

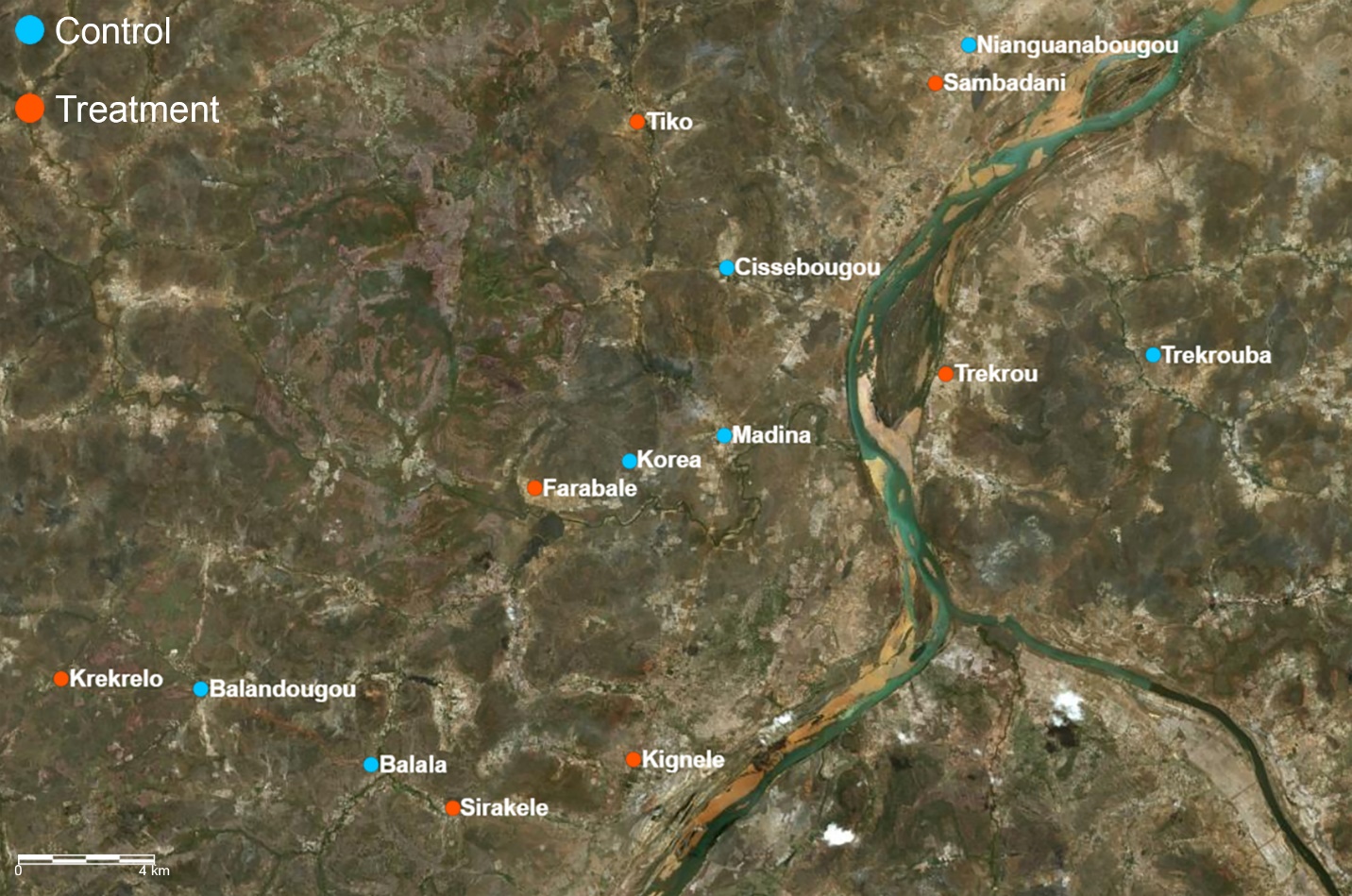


**Additional file 1: Fig. S1.** Map of the positions of the 14 study villages and their relation to the Niger River. Generated by Google Maps Professional.
