# Supplementary material for: Large-scale field trial of attractive toxic sugar baits (ATSB) for the control of malaria vector mosquitoes in Mali, West Africa
Source: Malar J. 2020 Feb 14;19:72. doi: 10.1186/s12936-020-3132-0 (PMC7023716; doi:10.1186/s12936-020-3132-0)
Supplement: Supplementary file 2 — Additional file 2: Fig. S2. A schematic presentation of bait station with a magnification of the permeable, black plastic membrane penetrable enough for mosquitoes to feed. B) Photo of the bait station hung on the outer wall of a house. [file 12936_2020_3132_MOESM2_ESM.docx]

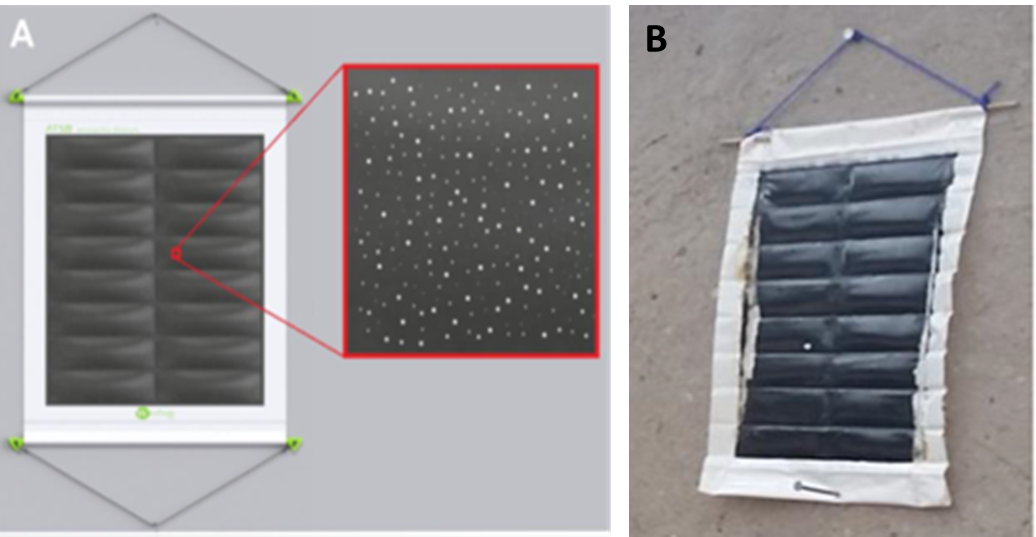


**Additional file 2: Fig. S2.** A Schematic presentation of bait station with a magnification of the permeable, black plastic membrane penetrable enough for mosquitoes to feed. B) Photo of the bait station hung on the outer wall of a house.
